# Comparative Antimicrobial and Antibiofilm Activity of Antiseptics and Commercial Mouthwashes Against *Porphyromonas gingivalis* ATCC 33277

**DOI:** 10.3390/jcm14248909

**Published:** 2025-12-17

**Authors:** Marzena Korbecka-Paczkowska, Tomasz M. Karpiński, Marcin Ożarowski

**Affiliations:** 1 Chair and Department of Medical Microbiology, Poznań University of Medical Sciences, Rokietnicka 10, 60-806 Poznań, Poland; mkorbecka@wp.pl; 2 Medi Pharm, os. Konstytucji 3 Maja 14/2, 63-200 Jarocin, Poland; 3 Department of Biotechnology, Institute of Natural Fibres and Medicinal Plants—National Research Institute, Wojska Polskiego 71B, 60-630 Poznań, Poland; marcin.ozarowski@iwnirz.pl

**Keywords:** planktonic growth, cell viability, biofilm destruction, periodontitis, gingivitis, treatment, prophylaxis

## Abstract

**Background:** *Porphyromonas gingivalis* is one of the most prevalent periodontal pathogens, involved in the development of periodontitis, deep caries, pulpitis, endodontic infections, and peri-implantitis. Antiseptics are commonly used in the treatment of oral diseases, but their effectiveness against *P. gingivalis* remains only partially understood. This preliminary study investigated antimicrobial and antibiofilm activity of eight pure antiseptics: boric acid (BA), chlorhexidine (CHX), ethacridine lactate (ET), hydrogen peroxide (H_2_O_2_), octenidine (OCT), polyhexanide (PHMB), potassium permanganate (KMnO_4_), and sodium hypochlorite (NaOCl), as well as five commercial rinses containing these agents, against periopathogen *P. gingivalis* ATCC 33277. **Methods:** Minimal inhibitory concentrations (MICs) were determined using the broth microdilution method. The Clinical Efficiency of MIC (CEMIC) was subsequently calculated. Antibiofilm activity was evaluated using the crystal violet method, LIVE/DEAD fluorescence assay and by measuring biofilm thickness with digital microscopy in combination with the author’s Python-based application Biofilm Thickness Analyzer. **Results:** OCT, CHX, PHMB and ET showed the strongest activity against *P. gingivalis*, in both its planktonic and biofilm forms. H_2_O_2_ and BA had variable MIC efficacy and moderate antibiofilm activity. In contrast, NaOCl and KMnO_4_ demonstrated the weakest activity or no significant effect against *P. gingivalis*. **Conclusions:** The results have a translational dimension, supporting the potential clinical relevance of the selected compounds. However, this study was conducted strictly in vitro on a single strain under monomicrobial biofilm conditions. Therefore, while the findings suggest that mouthwashes containing OCT, CHX, and PHMB may be effective against *P. gingivalis*, their actual clinical efficacy in the treatment and prevention of oral diseases remains to be confirmed in in vivo studies.

## 1. Introduction

Periodontal diseases represent a significant public health concern. According to available data, plaque-induced gingivitis affects between 90% and 100% of the adult population [1,2,3], while periodontitis occurs in approximately 60% of adults [4,5]. *Porphyromonas gingivalis* is one of the primary etiological agents responsible for both gingivitis and periodontitis in patients with subgingival plaque. It belongs to the so-called “red complex” of bacteria, which is strongly associated with the progression of periodontitis and includes of *P. gingivalis*, *Treponema denticola*, and *Tannerella forsythia* [6].

*P. gingivalis* is a Gram-negative, anaerobic bacterium that possesses numerous virulence factors. Many strains produce a capsule, a polysaccharide-rich outer structure, also referred to as the K-antigen. It protects the bacterial cell from phagocytosis and intracellular killing [7]. Additionally, the capsule can bind to other periopathogens, promoting bacterial co-aggregation and biofilm formation [8]. *P. gingivalis* produces two types of fimbriae: long fimbriae, composed of FimA protein subunits, and short fimbriae, composed of Mfa1 protein subunits. These structures facilitate interactions with other oral bacteria, promote biofilm formation, and enable invasion of host cells [9]. The long fimbriae interact with Toll-like receptor 2 (TLR2), triggering an inflammatory response characterized by the activation and upregulation of pro-inflammatory cytokines, particularly interleukin-8 (IL-8), tumor necrosis factor-alpha (TNF-α), and nuclear factor kappa B (NF-κB). The short fimbriae contribute to osteoclastogenesis and stimulate the production of IL-1β, IL-6, and TNF-α, ultimately promoting alveolar bone resorption [7,10]. Lipopolysaccharide (LPS), another key virulence factor, acts as a microbe-associated molecular pattern (MAMP) and, through structural variations in its lipid A component, activates specific Toll-like receptors (TLR2 and TLR4) on host cells [7,10]. This activation results in the induction of pro-inflammatory cytokines such as IL-1β, IL-6, and TNF-α, further contributing to alveolar bone resorption [11]. Another critical virulence determinant of *P. gingivalis* is the family of gingipains, surface-associated cysteine proteases responsible for the bacterium’s proteolytic activity. These enzymes are categorized into arginine-specific (RgpA and RgpB) and lysine-specific (Kgp) types [12]. Gingipains degrade extracellular matrix components, facilitate biofilm formation, and enhance bacterial adhesion and coaggregation. Moreover, they impair host immune responses by degrading defensins, complement proteins, and adhesion molecules, resulting in persistent inflammation and progressive destruction of periodontal tissues [12,13]. As described above, multiple virulence factors, including capsules, fimbriae, and gingipains, play pivotal roles in biofilm formation. The development and maturation of biofilm on tooth surfaces and within periodontal pockets are key processes driving the initiation and progression of periodontitis [11]. Outer membrane vesicles (OMVs) produced by *P. gingivalis* also contribute significantly to inter-bacterial communication and interactions with host cells. These vesicles facilitate the delivery of virulence factors into host tissues, exacerbating inflammation and tissue destruction. OMVs have additionally been implicated in the pathogenesis of various systemic diseases [14]. This is supported by studies demonstrating associations between periodontal pathogens and conditions such as rheumatoid arthritis [15], abdominal aortic aneurysm [16], arterial hypertension, diabetes [17], inflammatory bowel diseases [18], Alzheimer’s disease, non-alcoholic fatty liver disease [14], as well as cancers of the oral cavity, esophagus, colorectum, and pancreas [19]. Furthermore, maternal periodontitis has been associated with an increased risk of preterm birth and/or low birth weight in neonates [20].

The treatment of periodontal diseases, particularly periodontitis, involves oral hygiene education, non-surgical interventions such as scaling and root planing, pharmacological therapy including non-steroidal anti-inflammatory drugs and antibiotics, and, when indicated, surgical procedures [21,22,23]. Additionally, antiseptics are commonly employed in the form of mouth rinses or gels to support mechanical debridement and reduce the microbial load in the oral cavity [24,25].

This preliminary study included an evaluation of five commercially available mouthwashes or rinses and eight individual antiseptic agents. Among the antiseptics tested, chlorhexidine digluconate (CHX), octenidine dihydrochloride (OCT), and polyhexamethylene biguanide (PHMB) are widely recognized and routinely incorporated into oral care formulations. In contrast, sodium hypochlorite (NaOCl) is primarily applied at high concentrations in endodontic procedures and at low concentrations for rinsing the oral cavity and skin wounds [26]. The remaining substances, boric acid (BA), ethacridine lactate (ET), hydrogen peroxide (H_2_O_2_), and potassium permanganate (KMnO_4_), are classified as “old antiseptics” and are no longer routinely recommended for intraoral or wound use [27,28]. Finally, the objective of this study was to evaluate the minimum inhibitory concentrations (MICs), the Clinical Efficiency of MIC (CEMIC), and the antibiofilm effects of antiseptics and mouthwashes containing these compounds against the periopathogen *P. gingivalis* ATCC 33277.

## 2. Materials and Methods

### 2.1. Antiseptics

In all tests, the concentrations of the active compounds reflected those found in current commercially available oral antiseptics or topical wound care products. A detailed overview of the tested substances, their initial concentrations, and manufacturers is provided in Table 1.

### 2.2. Porphyromonas gingivalis Strain

The *P. gingivalis* reference strain (ATCC 33277) was obtained from Argenta (Poznań, Poland). Cultivation was carried out in Schaedler Broth and on Schaedler Agar enriched with 5% defibrinated sheep blood (Graso Biotech, Starogard Gdański, Poland). The cultures were incubated under anaerobic conditions at 36 °C for 3 to 5 days.

### 2.3. Minimal Inhibitory Concentrations (MIC)

The minimum inhibitory concentrations (MICs) of the tested antiseptics were assessed using a microdilution technique in sterile 96-well microplates (Nest Scientific Biotechnology, Wuxi, China) [29]. Specifically, Schaedler Broth (Graso Biotech, Starogard Gdański, Poland) was used as the culture medium, and each well contained a final volume of 200 µL. Two-fold serial dilutions of the antiseptics were prepared, beginning from the initial concentrations specified in Table 1. The inoculated plates were then incubated under anaerobic conditions at 36 °C for 48 h. After incubation, MIC endpoints were determined by visual inspection, and in ambiguous cases, confirmed by adding 10 µL of a 1% aqueous solution of 2,3,5-triphenyl-tetrazolium chloride (TTC) (Sigma-Aldrich, Poznań, Poland) to facilitate colorimetric detection of bacterial viability.

### 2.4. Clinical Efficiency of MIC (CEMIC)

To assess the clinical applicability of the MIC values obtained, the Clinical Efficiency of MIC (CEMIC) index was used. This index was determined by calculating the ratio between the minimum inhibitory concentration and the standard therapeutic concentration of the antimicrobial agent, using the following formula [30]:

$$Clinical\;Efficiency\;of\;MIC\;(CEMIC)=\frac{MIC}{Clinical\;concentration}$$

Interpretation of the results was based on the following criteria:

- CEMIC < 0.1 indicated high clinical efficiency,
- values between 0.1 and 0.9 were considered to reflect moderate efficiency,
- while values > 0.9 suggested low clinical usefulness of the compound at the tested concentration.

### 2.5. Minimal Bactericidal Concentration (MBC)

The minimal bactericidal concentrations (MBCs) were assessed by transferring 10 µL of the suspensions obtained from the MIC assay onto Schaedler agar plates. The MBC was considered the lowest concentration of a given antiseptic or mouthwash at which no viable colonies appeared. To differentiate between bactericidal and bacteriostatic activity, the MBC/MIC ratio was applied. A value of ≤4 was interpreted as indicative of a bactericidal effect, whereas ratios of ≥8 were classified as evidence of a bacteriostatic mechanism [31].

### 2.6. Antibiofilm Effects

#### 2.6.1. Crystal Violet Method

The aim of this assay was to determine the minimal effective exposure time for antibiofilm activity, which was assessed using the crystal violet method. The methodology has been described in a previous publication [31]. Shortly, mature biofilms were formed in 96-well plates with Schaedler Broth, using bacterial suspensions adjusted to 0.5 McFarland, and incubated anaerobically at 36 °C for 72 h. Wells were then treated with 100 µL of commercial concentrations of antiseptics or mouthwashes for 1, 10, 30, and 60 min. After treatment, biofilms were fixed with methanol, stained with 1% crystal violet, and the dye was subsequently solubilized with 96% ethanol. Biofilm biomass was quantified by measuring optical density at 630 nm using a microplate reader (ELISA 250, BioMerieux, Warsaw, Poland). All experiments were performed in duplicate. The strongest antibiofilm effect was observed after 1 h of exposure, which was used for all subsequent assays.

#### 2.6.2. Biofilm Thickness Analysis

Antibiofilm activity was evaluated using 12-well culture plates (Nest Scientific Biotechnology, Wuxi, China). Biofilms were formed by inoculating each well with 1 mL of bacterial suspension prepared in Schaedler Broth and adjusted to a 0.5 McFarland standard. Plates were incubated anaerobically at 36 °C for 72 h to allow for mature biofilm development. After incubation, wells were rinsed three times with sterile 0.9% NaCl to remove planktonic cells. Subsequently, 1 mL of each antiseptic or mouthwash solution, used at its commercial concentration (as detailed in Table 1), was added to the biofilms. The contact time was 1 h at 36 °C. After exposure, residual antiseptics were removed and neutralized by treating the wells with alkaline saline peptone water (Sigma-Aldrich, Poznań, Poland) for 5 min, followed by another rinse with 0.9% NaCl.

Biofilm thickness was analyzed using a Keyence VHX-S770E digital microscope (Keyence International, Mechelen, Belgium). A three-dimensional reconstruction of the biofilm surface was generated, and image data were further analyzed using an author’s Python-based application, Biofilm Thickness Analyzer [32], designed to calculate average biofilm thickness for each image and perform basic statistical assessments based on 3D image input.

#### 2.6.3. LIVE/DEAD Biofilm Viability

Viability of *P. gingivalis* was assessed by staining with the LIVE/DEAD™ BacLight™ Bacterial Viability Kit (Invitrogen, Waltham, MA, USA), according to the manufacturer’s manual. This staining kit uses a mixture of the green-fluorescent nucleic acid stain SYTO^®^ 9 and the red-fluorescent nucleic acid stain propidium iodide. Live cells were stained green, whereas dead cells with damaged membranes were stained red [33]. Fluorescence microscopy was performed using a Leica DM1000 microscope (Leica, Wetzlar, Germany), and images were captured with a Progres Gryphax camera (Jenoptik AG, Jena, Germany). For each sample, the numbers of live and dead bacteria were manually counted in three randomly selected microscopic fields to assess cell viability.

### 2.7. Statistics

One-way ANOVA with Tukey post-tests was used to assess the statistical significance of differences in the biofilm mass, viability and thickness. Results were considered significant at a *p*-value of less than 0.05. Data analysis was performed using InStat 3.10 software (GraphPad Software, Boston, MA, USA).

## 3. Results

### 3.1. Antimicrobial Activity (MIC, MBC and CEMIC)

In the microdilution method the best activity against the planktonic *P. gingivalis* was demonstrated by OCT and CHX. Their MIC values were below 1 µg/mL for OCT and below 1 µg/mL up to 2 µg/mL for CHX, which was 500–1000 times lower than the commercial concentration. High activity against periopathogen was also shown by H_2_O_2_ and PHMB, with MIC values more than 60 times lower than the commercial concentrations. BA had weaker activity, working only in several-fold dilutions. NaOCl and KMnO_4_ were active only at their commercial concentrations. Similar results to those obtained for the pure compounds were observed for the commercial products containing OCT, CHX, PHMB, or NaOCl/HOCl (Table 2).

The lowest CEMIC values, indicating excellent clinical efficiency, were observed for formulations containing OCT, CHX, and PHMB. Pure compounds BA and H_2_O_2_ showed moderate CEMIC values, whereas products containing NaOCl and KMnO_4_ exhibited low clinical usefulness. At the same time, all pure antiseptics and mouthwashes exhibited bactericidal activity. Detailed results are presented in Table 2.

### 3.2. Cell Viability and Antibiofilm Activity

**Figure 1:**
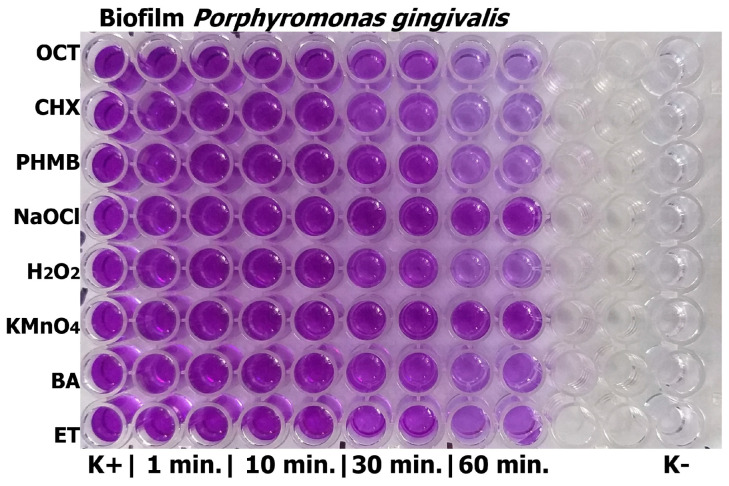
Results of crystal violet tests of pure antiseptics on the *Porphyromonas gingivalis* biofilm. OCT—octenidine dihydrochloride; CHX—chlorhexidine digluconate; PHMB—polyhexamethylene biguanide (polyhexanide); NaOCl—sodium hypochlorite; H_2_O_2_—hydrogen peroxide; KMnO_4_—potassium permanganate; BA—boric acid; ET—ethacridine lactate; K+—positive control with bacteria; K(−)—negative control (only Schaedler Broth).

The crystal violet method was used as a screening approach to determine the minimal effective exposure time for most of the tested substances. The results obtained for exposure times of 1, 10, and 30 min did not differ significantly from the control and are therefore not presented in this study. The significantly important antibiofilm effect was observed after 1 h of exposure for OCT, CHX, PHMB, H_2_O_2_, and ET. Based on these findings, an exposure time of 1 h was adopted for all subsequent assays. The microplate layout for this experiment is shown in Figure 1, and the absorbance values obtained after 1 h of incubation are presented in Table 3.

The control group exhibited approximately 99% viability of *P. gingivalis* and served as the reference point for evaluating the effects of all tested antiseptics. Among the pure compounds, after one hour of incubation, OCT, H_2_O_2_, and NaOCl significantly reduced bacterial viability by more than 50% compared with the control (*p* < 0.001), namely 57.3%, 58.7% and 74%. PHMB, ET and BA decreased viability by 47.7%, 46% and 41.3% on average, also showing a highly significant difference from the control (*p* < 0.001). CHX caused a moderate reduction in viability, averaging just under 20% compared with the control, which was statistically significant at the level of *p* < 0.05. KMnO_4_ was the only compound that did not produce significant changes in bacterial viability. For commercial mouthwashes, all formulations containing OCT, CHX, PHMB, and NaOCl exhibited a significant reduction in *P. gingivalis* viability.

In the biofilm thickness analysis after one hour of exposure, the control sample exhibited the greatest thickness, averaging 636 ± 321 µm. Pure NaOCl, and KMnO_4_ did not produce significant reductions in biofilm thickness, which remained around 600 µm, similar to the control. No significant reductions were observed for BA or H_2_O_2_ either, with mean biofilm thicknesses of 303 µm and 349 µm, respectively. In contrast, OCT, CHX, PHMB, their corresponding commercial mouthwashes and ET, significantly reduced biofilm thickness to values about or below 200 µm.

The combined analysis of three parameters, biofilm mass, thickness and bacterial viability, provides a comprehensive assessment of the efficacy of the tested antiseptics. OCT, CHX, PHMB and ET demonstrated the strongest antimicrobial and antibiofilm activities. In contrast, preparations containing BA, H_2_O_2_, and NaOCl exhibited substantial bactericidal effects but lacked antibiofilm activity, suggesting limited penetration into the biofilm and poor ability to disrupt its extracellular matrix. The weakest performance was observed for KMnO_4_, which showed no significant effects in either of the analyzed parameters.

**Figure 2:**
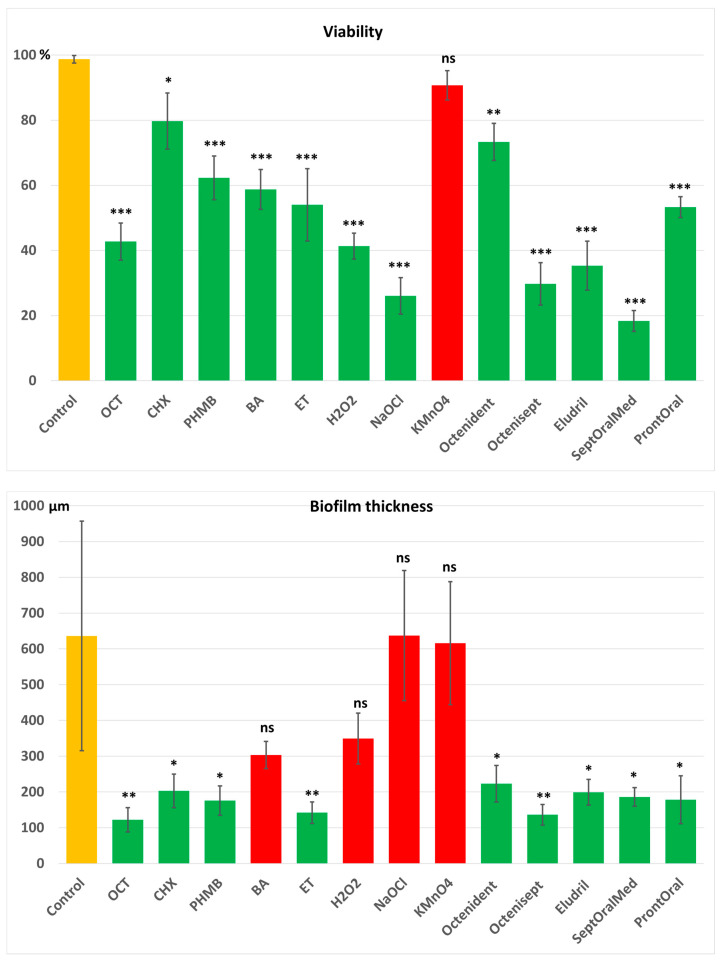
Results of 1 h exposure of the tested antiseptics and mouthwashes on the *Porphyromonas gingivalis* viability and biofilm thickness. Statistical difference compared to the control: ns—not statistically significant; *—*p* < 0.05; **—*p* < 0.01; ***—*p* < 0.001. OCT—octenidine dihydrochloride; CHX—chlorhexidine digluconate; PHMB—polyhexamethylene biguanide (polyhexanide); BA—boric acid; ET—ethacridine lactate; NaOCl—sodium hypochlorite; H_2_O_2_—hydrogen peroxide; KMnO_4_—potassium permanganate.

**Figure 3:**
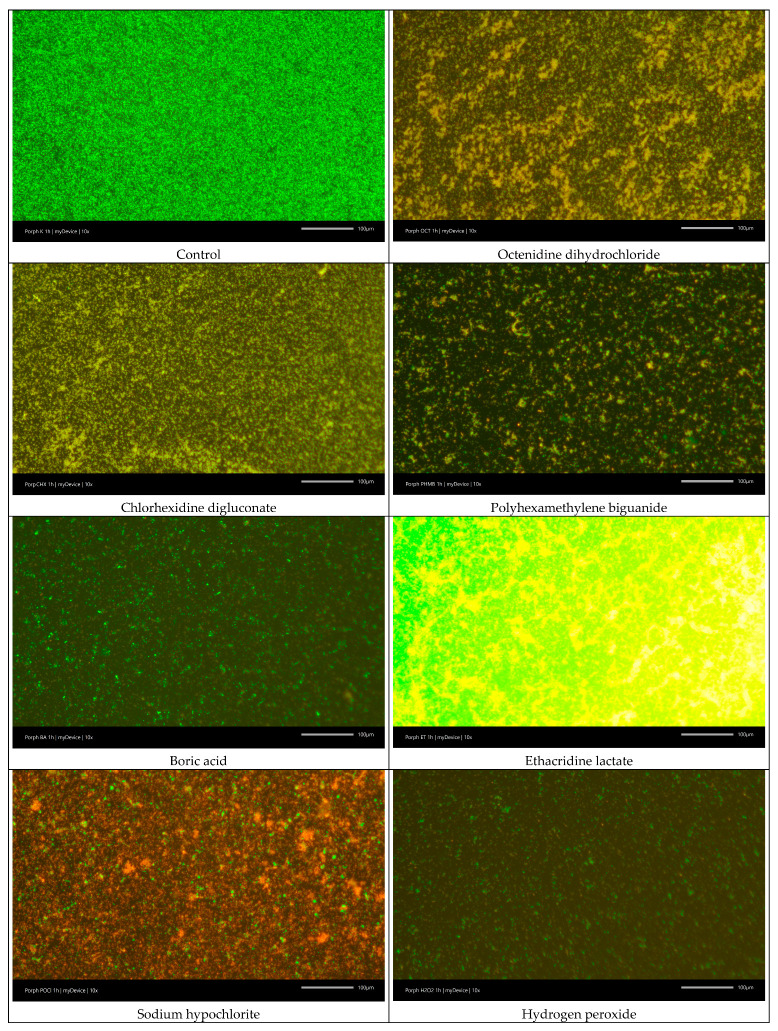

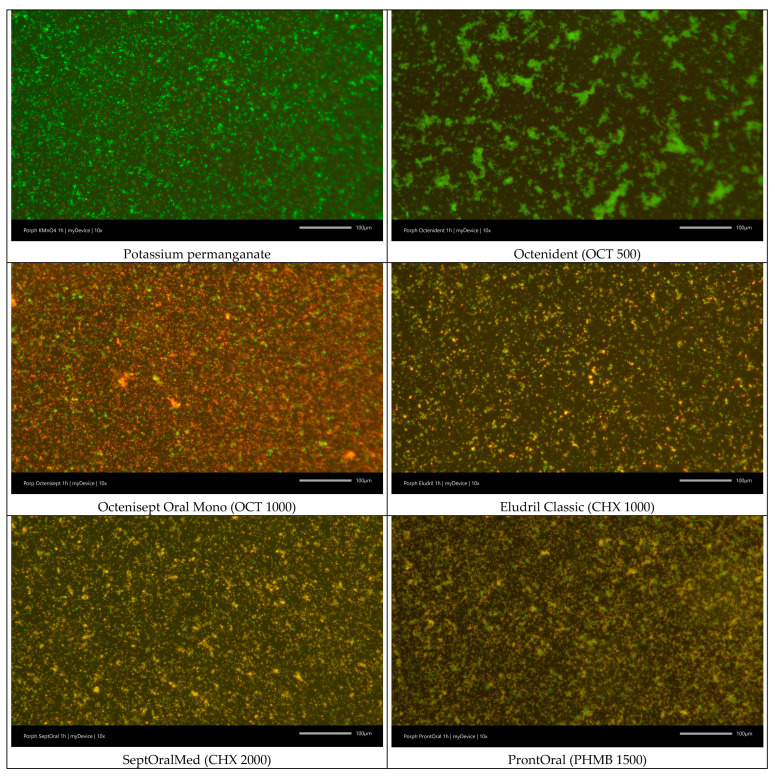
Sample photos of *Porphyromonas gingivalis* biofilms viability assay, after 1 h incubation with pure antiseptics and commercial mouthwashes. To assess bacterial viability, the LIVE/DEAD™ BacLight™ Bacterial Viability Kit, a fluorescence-based assay, was used. This method differentiates live and dead cells based on membrane integrity. Live bacteria with intact membranes are stained green, whereas cells with compromised membranes take up a red fluorescent dye.

**Figure 4:**
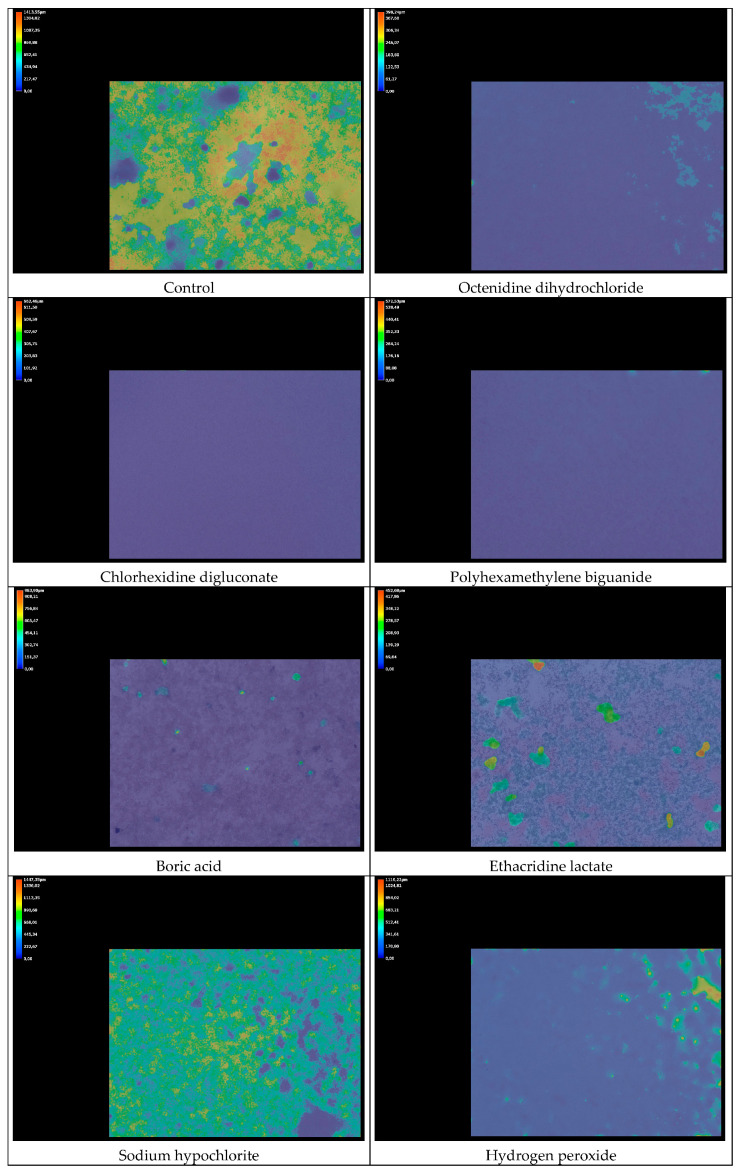

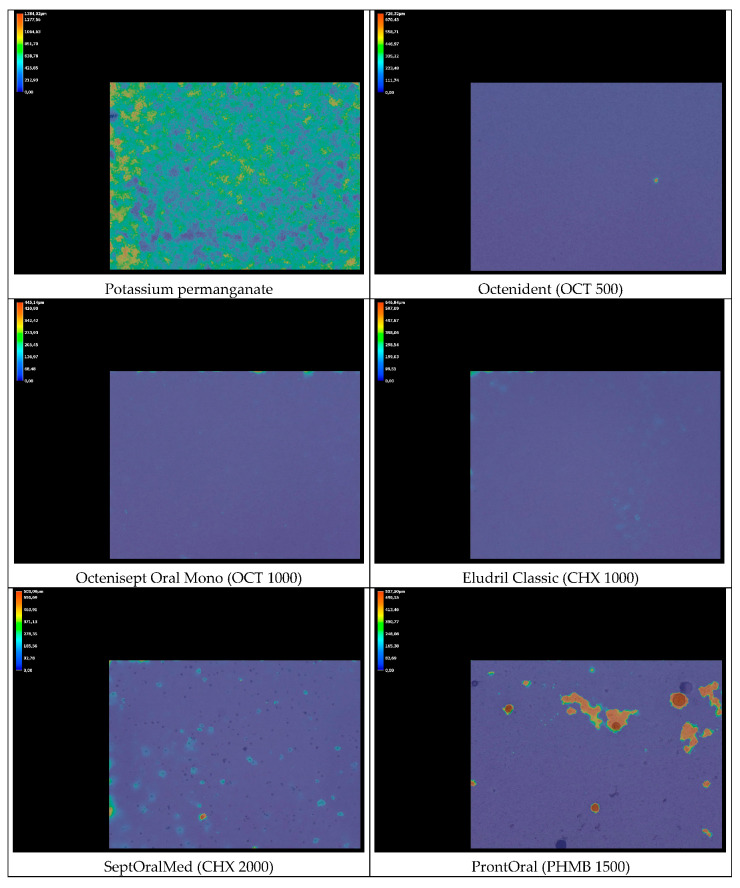
Sample photos of *Porphyromonas gingivalis* biofilms thickness assessment after 1 h incubation with pure antiseptics and commercial mouthwashes. The color scale on the upper left of the images represents biofilm thickness in micrometers (µm). The scale ranges from dark blue for the thinnest regions to red for the thickest areas. Intermediate colors, light blue, green, yellow, and orange, indicate progressively increasing biofilm height. The average biofilm thickness for each image was assessed using the Python-based application Biofilm Thickness Analyzer [32].

The results are presented in Figure 2, and representative images showing bacterial viability and biofilm thickness after 1 h of antiseptics exposure are in Figure 3 and Figure 4, respectively.

Table 4 summarizes the activity of antiseptics against *P. gingivalis*, considering both clinical efficacy and antibiofilm properties. In oral infections, biofilm formation plays a central pathogenic role, making antibiofilm activity the most important criterion for interpreting the results. Following this approach, OCT, CHX, PHMB and ET demonstrated the strongest activity. Comparable outcomes were obtained for the commercial formulations containing these compounds. Simultaneously, H_2_O_2_ and BA showed limited effect against *P. gingivalis*. In contrast, NaOCl and KMnO_4_ lack clinical utility and are not recommended for use in oral infections.

## 4. Discussion

In the present study, the most potent activity against *P. gingivalis* was observed for OCT and CHX, both exhibiting minimum inhibitory concentrations (MICs) ≤ 2 µg/mL. Literature data indicate that OCT and CHX display comparable MIC values against other periodontal pathogens. OCT demonstrated an MIC of 0.12 µg/mL against *Aggregatibacter actinomycetemcomitans* [34], while the MICs of CHX against *Actinomyces israelii*, *A. actinomycetemcomitans*, *Fusobacterium nucleatum*, *P. gingivalis*, *Prevotella endodontalis*, and *P. intermedia*, and ranged from 1.6 to 4.0 µg/mL [35,36,37]. However, some studies have reported substantially higher MICs for CHX against periodontal anaerobes, ranging from 31.7 to 62.9 µg/mL [38] and even 100 µg/mL [39]. In biofilm-forming conditions, CHX activity decreases markedly, with reported MICs of 128–256 µg/mL for *P. gingivalis*, 256–512 µg/mL for *P. intermedia*, and 1024 µg/mL for *A. israelii* [40]. High activity against *P. gingivalis* was also observed for H_2_O_2_ and PHMB. Lee reported an MIC of 0.1% (1000 µg/mL) for H_2_O_2_ against *P. gingivalis* [39], whereas in the present study, this value was approximately twofold lower.

With respect to the rate of action, a 90% reduction in *P. gingivalis* viability was observed after 15 min of exposure, and complete eradication after 30 min when treated with Corsodyl, a commercial mouthwash containing 0.2% (2000 µg/mL) CHX [41]. In contrast, Acclean, containing 0.12% CHX, reduced the viability of anaerobic bacteria, including *P. gingivalis*, within a biofilm by approximately 80% after only 10 min of exposure [42]. In our experiments, pure 0.1% CHX induced only a moderate reduction in bacterial viability after one hour, whereas commercial formulations achieved over 60% reduction, likely due to the presence of auxiliary ingredients enhancing antimicrobial efficacy.

BA exhibited comparatively weak activity, with an MIC of 3750 µg/mL. Previous studies have reported MICs ranging from 770 to 3090 µg/mL against aerobic bacteria such as *Staphylococcus aureus*, *Aeromonas hydrophila*, and *Pseudomonas aeruginosa* [43]. In a clinical trial, BA at 7500 µg/mL significantly reduced the prevalence of *A. actinomycetemcomitans* and *F. nucleatum* in patients with periodontitis [44].

NaOCl and KMnO_4_ demonstrated antimicrobial activity only at their commercial concentrations, with even twofold dilutions showing no effect. Reported MIC values for NaOCl vary considerably. Sisodiya et al. observed an MIC of 45 µg/mL against *F. nucleatum* [45], whereas other studies reported values between 330 and 780 µg/mL against periodontal pathogens such as *A. actinomycetemcomitans*, *F. nucleatum*, *P. gingivalis*, and *Prevotella* spp. [36,38]. Numerous studies confirming the antimicrobial efficacy of NaOCl employed substantially higher concentrations, typically ranging from 22,500 to 30,000 µg/mL [46,47]. However, recent findings indicate that NaOCl and KMnO_4_ possess a high Karpinski Adaptation Index (KAI), suggesting a considerable potential for the rapid development of microbial resistance [30,48,49].

According to current guidelines, BA, KMnO_4_, and H_2_O_2_ are regarded as outdated antiseptics and are not recommended, for example, in wound care, due to their cytotoxic effects, limited antimicrobial efficacy, and potential to promote resistance [27,28]. Our findings confirm the limited antibiofilm activity of these compounds and the weak planktonic activity of KMnO_4_ against *P. gingivalis*, supporting the rationale for avoiding their use in oral infection management.

## 5. Limitations

This study has several limitations. First, all experiments were conducted in vitro, which does not fully replicate the complex environmental conditions of the oral cavity. Factors such as salivary flow, enzymatic degradation, immune response, and multispecies microbial interactions, each of which may influence antiseptic efficacy, were not represented. In particular, the antibiofilm exposure time (1 h) does not reflect the actual mouthwash contact time in patients (~30–60 s), which suggests that the observed effects in this study may overestimate the efficacy of the antiseptics under real-use conditions. Consequently, the clinical performance of the tested agents may differ from the laboratory results. Second, the research was performed using a single reference strain, *P. gingivalis* ATCC 33277. Although this strain is widely utilized in antimicrobial studies [50,51,52], clinical isolates may exhibit strain-dependent variability in susceptibility and biofilm formation. Nevertheless, our previous studies have demonstrated that antiseptic activity among strains of the same species tends to be comparable [30,49]. Similar observations have been reported by other authors, who found minimal MIC variations among one species strains, including *Staphylococcus* spp., *Enterococcus* spp., *Klebsiella pneumoniae*, *Escherichia coli*, *P. aeruginosa*, *Enterobacter cloacae*, *Acinetobacter baumannii*, *Candida albicans*, and oral anaerobes. These variations, also observed between species, typically amounted to only one dilution step for small dilutions and up to three dilution steps for larger dilutions [53,54,55,56,57,58].

Future studies incorporating multiple clinical isolates would enable a more comprehensive assessment of variability in antiseptic susceptibility within *P. gingivalis* populations. However, the isolation of *P. gingivalis* from clinical samples poses substantial challenges. This obligate anaerobe requires strictly controlled growth conditions and prolonged incubation, which complicate the maintenance of viable isolates. Furthermore, the collection of subgingival biofilm material is technically demanding due to possible low abundance of *P. gingivalis* within complex multispecies communities [59]. Consequently, selective culture media and molecular methods such as matrix-assisted laser desorption/ionization time-of-flight mass spectrometry (MALDI-TOF MS) or 16S rRNA gene sequencing are required to ensure isolate purity and accurate identification [60]. For these reasons, many studies utilize only one reference strain of *P. gingivalis* [61,62,63,64].

## 6. Conclusions

Octenidine, chlorhexidine, polyhexanide and ethacridine lactate demonstrated the highest antimicrobial activity against *Porphyromonas gingivalis* in both planktonic and biofilm forms. Notably, commercial mouthwashes containing OCT, CHX and PHMB also exhibited strong inhibitory effects.

In contrast, antiseptics such as hydrogen peroxide and boric acid showed variable efficacy based on their MIC values and moderate antibiofilm activity. Sodium hypochlorite and potassium permanganate had the poorest activity. From a clinical perspective, these compounds are therefore not recommended for the management of oral infections caused by *P. gingivalis*.

Overall, this study provides valuable translational insight by identifying antiseptics with potent activity against *P. gingivalis*, thus informing potential practical dental applications. However, as the experiments were conducted in vitro on a single *P. gingivalis* strain, the clinical relevance of these findings remains to be confirmed. The results suggest that OCT-, CHX-, and PHMB-based mouthwashes may be effective against *P. gingivalis*, but their actual efficacy in the treatment and prevention of periodontal diseases, endodontic infections, or peri-implantitis requires further in vivo validation. These findings provide a foundation for future evidence-based studies on antiseptic use in modern dental practice.

## Figures and Tables

**Table 1:** Concentrations of pure antiseptics, and active compounds in mouthwashes used in the studies.

Pure Antiseptics	Used Concentration (µg/mL)
Octenidine dihydrochloride (OCT) (Schülke & Mayr GmbH, Norderstedt, Germany)	1000
Chlorhexidine digluconate (CHX) (Sigma-Aldrich, Poznań, Poland)	1000
Polyaminopropyl biguanide (Polihexanide, PHMB) (Arxada AG, Basel, Switzerland)	1000
Boric acid (BA) (Herbapol, Poznań, Poland)	30,000
Ethacridine lactate (ET) (Herbapol, Poznań, Poland)	1000
Sodium hypochlorite (NaOCl) (Cerkamed, Stalowa Wola, Poland)	100
Hydrogen peroxide (H_2_O_2_) (Hasco-Lek S.A., Wrocław, Poland)	30,000
Potassium permanganate (KMnO_4_) (Hasco-Lek S.A., Wrocław, Poland)	10,000
**Mouthwashes**	**Active Compound and Its Concentration (µg/mL)**
Octenident^®^ (Schülke & Mayr GmbH, Norderstedt, Germany)	OCT 500
Octenisept Oral Mono^®^ (Schülke & Mayr GmbH, Norderstedt, Germany)	OCT 1000
Eludril Classic^®^ (Pierre Fabre, Cahors, France)	CHX 1000
SeptOralMed^®^ (Avec Pharma, Wrocław, Poland)	CHX 2000
ProntOral^®^ (B Braun, Melsungen, Germany)	PHMB 1500

**Table 2:** Ranges of minimal inhibitory concentration (MIC), minimal bactericidal concentrations (MBC) and Clinical Efficiency of MIC (CEMIC) values for antiseptics and mouthwashes against *Porphyromonas gingivalis*.

Antiseptic/Mouthwash	MICs of the Product [%]	MICs/MBCs of Active Compound [µg/mL]	CEMIC	MBC/MIC Ratio
Octenidine dihydrochloride (OCT) 1000 µg/mL	0.024–0.049	0.24–0.49	0.00024–0.00049	1
Chlorhexidine digluconate (CHX) 1000 µg/mL	0.098–0.195	0.98–1.95	0.00098–0.00195	1
Polyhexamethylene biguanide (PHMB) 1000 µg/mL	0.781–1.56	7.81–15.63	0.00781–0.0156	1
Boric acid (BA) 30,000 µg/mL	12.5	3750	0.125	1
Ethacridine lactate (ET) 1000 µg/mL	6.25	62.5	0.00625	1
Hydrogen peroxide (H_2_O_2_) 30,000 µg/mL	1.56	470	0.0157	1
Sodium hypochlorite (NaOCl) 200 µg/mL	100	100/200	1	2
Potassium permanganate (KMnO_4_) 10,000 µg/mL	100	10,000	1	1
Octenident^®^ (OCT 800 µg/mL)	0.098	0.78	0.00098	1
Octenisept Oral Mono^®^ (OCT 1000 µg/mL)	0.024–0.049	0.24–0.49	0.00024–0.00049	1
Eludril Classic^®^ (CHX 1000 µg/mL)	0.049–0.098	0.49–0.98	0.00049–0.00098	1
SeptOralMed^®^ (CHX 2000 µg/mL)	0.024	0.49	0.000245	1
ProntOral^®^ (PHMB 1500 µg/mL)	0.781	11.72	0.0078	1

CEMIC results were interpreted as: <0.1—excellent efficiency; 0.1–0.9—moderate; >0.9—low clinical usefulness at the tested concentration [30].

**Table 3:** Absorbance values (OD at 630 nm) corresponding to biofilm reduction in the crystal violet assay after 1 h exposure time.

Antiseptic	Absorbance After 1 h Exposure Time
Control	0.841 ± 0.033
OCT 1000 µg/mL	0.172 ± 0.033 ***
CHX 1000 µg/mL	0.196 ± 0.028 ***
PHMB 1000 µg/mL	0.260 ± 0.036 ***
BA 30,000 µg/mL	0.708 ± 0.033 ns
ET 1000 µg/mL	0.553 ± 0.031 **
H_2_O_2_ 30,000 µg/mL	0.405 ± 0.053 ***
NaOCl 200 µg/mL	0.766 ± 0.042 ns
KMnO_4_ 10,000 µg/mL	0.790 ± 0.063 ns

OCT—octenidine dihydrochloride; CHX—chlorhexidine digluconate; PHMB—polyhexamethylene biguanide (polyhexanide); BA—boric acid; ET—ethacridine lactate; H_2_O_2_—hydrogen peroxide; NaOCl—sodium hypochlorite; KMnO_4_—potassium permanganate. Statistical difference compared to the control: ns—not statistically significant; **—*p* < 0.01; ***—*p* < 0.001.

**Table 4:** Summary of the antiseptics activity against *Porphyromonas gingivalis*.

Antiseptic	Clinical Efficiency of MIC (CEMIC)	Significant Reduction in Biofilm			Clinical Utility Against *P. gingivalis*
		Mass	Viability	Thickness	
Octenidine dihydrochloride (OCT) 1000 µg/mL	Excellent	Yes	Yes	Yes	Yes
Chlorhexidine digluconate (CHX) 1000 µg/mL	Excellent	Yes	Yes	Yes	Yes
Polyhexamethylene biguanide (PHMB) 1000 µg/mL	Excellent	Yes	Yes	Yes	Yes
Ethacridine lactate (ET) 1000 µg/mL	Excellent	Yes	Yes	Yes	Yes
Hydrogen peroxide (H_2_O_2_) 30,000 µg/mL	Excellent	Yes	Yes	No	Partially
Boric acid (BA) 30,000 µg/mL	Moderate	No	Yes	No	Partially
Sodium hypochlorite (NaOCl) 200 µg/mL	Poor	No	Yes	No	No
Potassium permanganate (KMnO_4_) 10,000 µg/mL	Poor	No	No	No	No

## Data Availability

The original contributions presented in the study are included in the article, further inquiries can be directed to the corresponding author.
